# Letter to the Editor, regarding Li L, Zhu X, Chen X, Gao J, Ding C, et al. Advances in targeted therapy for pulmonary arterial hypertension in children. Eur J Pediatr. 2022 Dec 17

**DOI:** 10.1007/s00431-023-04877-6

**Published:** 2023-02-27

**Authors:** Maurice Beghetti, Dunbar Ivy, Nicola Scott, Sandra Machlitt-Northen, Mary Ann Lukas

**Affiliations:** 1 Centre Universitaire de Cardiologie et Chirurgie Cardiaque Pédiatrique, Congenital Heart Center (CHUV et HUG), Geneva, Switzerland; 2 University of Colorado School of Medicine Children’s Hospital Colorado, Aurora, CO USA; 3 GlaxoSmithKline, Brentford, UK

We congratulate Li and colleagues on their comprehensive review on pulmonary arterial hypertension (PAH) therapy advances in children [1]. However, we note with concern that information provided on Volibris (ambrisentan) in Table 1 is not current for the European Union (EU) and Japan, with potential safety/dosing implications. Firstly, under “approved use,” it states “off-label” for ambrisentan, whereas regulatory approval was obtained in 2021 (September for the EU and April for Japan).

This is particularly important because the approved age is only from 8 to < 18 years, which is not detailed in the manuscript and the dosing information in the table does not reflect approved recommendations for dosing by weight [2]. The approval for ambrisentan only covers children weighing ≥ 20 kg with initial dosing of 2.5 mg (for which a tablet strength is now available), and up-titration dependent on clinical response and tolerability to 5 mg for children < 35 kg, or initial dose of 5 mg for weights ≥ 35 kg, with up-titration to 7.5 mg for 35 to < 50 kg, and to 10 mg for ≥ 50 kg. These data are based on the randomized clinical trial conducted by Ivy et al. [3], which is also referenced in the recent ESC/ERS guideline PAH update [4]. Lower dosing is recommended with cyclosporine A coadministration [2]. Lastly, safety and efficacy have not been established in patients under 8 years; hence, use in these patients is not recommended and as described in the EU product information [2], and by Laffan et al. [5], there may also be potential risk for ambrisentan in children < 4 years based on preclinical data in juvenile rats.

We hope this important corrective information is well received and that an updated version reflecting these updates will be published to provide practitioners with the appropriate and approved approach to ambrisentan use in children with PAH.

## Data Availability

Not applicable.
